# Skin-on-a-Chip: Transepithelial Electrical Resistance and Extracellular Acidification Measurements through an Automated Air-Liquid Interface

**DOI:** 10.3390/genes9020114

**Published:** 2018-02-21

**Authors:** Frank A. Alexander, Sebastian Eggert, Joachim Wiest

**Affiliations:** 1 cellasys GmbH, 87758 Kronburg, Germany; alexander@cellasys.com; 2 Department of Mechanical Engineering, Technical University of Munich, 80333 Munich, Germany; 3 Centre in Regenerative Medicine, Institute of Health and Biomedical Innovation, Queensland University of Technology, 4059 Kelvin Grove, Australia; s.eggert@qut.edu.au

**Keywords:** TEER, Organ-on-a-Chip, skin models, reconstructed human epidermis, impedance, label-free monitoring

## Abstract

Skin is a critical organ that plays a crucial role in defending the internal organs of the body. For this reason, extensive work has gone into creating artificial models of the epidermis for in vitro skin toxicity tests. These tissue models, called reconstructed human epidermis (RhE), are used by researchers in the pharmaceutical, cosmetic, and environmental arenas to evaluate skin toxicity upon exposure to xenobiotics. Here, we present a label-free solution that leverages the use of the intelligent mobile lab for in vitro diagnostics (IMOLA-IVD), a noninvasive, sensor-based platform, to monitor the transepithelial electrical resistance (TEER) of RhE models and adherent cells cultured on porous membrane inserts. Murine fibroblasts cultured on polycarbonate membranes were first used as a test model to optimize procedures using a custom BioChip encapsulation design, as well as dual fluidic configurations, for continuous and automated perfusion of membrane-bound cultures. Extracellular acidification rate (EAR) and TEER of membrane-bound L929 cells were monitored. The developed protocol was then used to monitor the TEER of MatTek EpiDerm^TM^ RhE models over a period of 48 h. TEER and EAR measurements demonstrated that the designed system is capable of maintaining stable cultures on the chip, monitoring metabolic parameters, and revealing tissue breakdown over time.

## 1. Introduction

As the largest organ of the body, skin represents an anatomical barrier between the external and internal environments of the human body, isolating internal organs from toxins and pathogens as well as protecting the underlying layers from ultraviolet (UV) radiation [1]. Besides the important barrier function, the human skin performs several essential functions of the human body, such as heat regulation, sensation, and excretion. Since the skin is the first protection shield of the human body against external environmental influences, new chemical formulations, such as drugs and toxins, have to be analyzed and evaluated for the ability to modulate skin integrity [2]. To investigate the influence of such compounds, cell biology utilizes cell culture models, providing a deeper understanding of the cellular behavior modulated by chemicals. Hence, within the last decade, a variety of bioengineered models have been developed for research on skin toxicity [3,4,5]. The first skin models were based on conventional two-dimensional (2D) co-culture models where keratinocytes were seeded onto pre-cultured fibroblasts [6]. Since culturing on rigid plastic does not maintain the epithelium for a longer period, and stratification of layers is prevented, the tissue engineering community developed 3D skin models to recapitulate the in vivo-like architecture and engineered a more physiologically relevant environment by integrating an air-liquid interface [7]. 

Human skin is composed of the following primary layers which carry out additional functions: The epidermis, the dermis, and the subcutaneous tissue. Depending on the integration of the different skin layers into the cellular model, current skin models are classified into reconstructed human epidermis models with keratinocytes, full thickness models with keratinocytes and fibroblasts representing dermal and epidermal compartments, and full thickness models with additional cell types (such as melanocytes, stem cells, and others) [8]. Due to their simplified architecture, reconstructed human epidermis (RhE) models have a high reproducibility and are therefore widely accepted. RhE models are made of human-derived keratinocytes seeded on semipermeable polycarbonate membranes, which are incorporated in a cell culture system placed into standard well plates, such as the commercially available Transwell^®^ systems (Corning Inc., Corning, NY, USA). The setup positions the culture at the air-liquid-interface, where the skin surface is exposed to the air, resulting in a physiologically relevant culture condition [9]. At present, there are open-source skin models available [10], as well as commercially available models, such as the EpiDerm™ (MatTek in vitro life science laboratories, Bratislava, Slovak Republic), EpiSkin™ (EpiSkin, Lyon Cedex, France), and SkinEthic™ (EpiSkin), EpiCS^®^ (Cell Systems GmbH, Troisdorf, Germany), and LabCyte (Japan Tissue Engineering Co., Ltd. [J-TEC], Aichi, Japan). Following a non-profit and patent-free approach, the Alternatives to Experiments on Animals Destined to Research Applications (ALEXANDRA) association offers protocols for fabrication and testing of open-source 3D skin models [11].

## 2. Background

### 2.1 State of the Art in Reconstructed Human Epidermis-Based Skin Toxicity Testing

To evaluate and predict the harmful effect of toxic compounds and drugs on the skin from a regulatory perspective, 3D model systems have to undergo extensive validation studies with a series of test compounds. Models with specified testing techniques are permitted as acceptable tools for skin toxicity testing, if their quality is proven according to European Center for Validation of Alternative Methods (ECVAM) standards and Organization of Economic Co-operation and Development (OECD) test guidelines (TG). Up to now, OECD provides guidelines describing skin corrosion and irritation testing based on OECD TG 431 and 439, respectively. 

### 2.2 Moving Toward Organ-on-Chip Platforms and Inclusion of Microphysiometry

As current models offer only limited significance in terms of biologic relevance, the field of toxicity testing is moving toward the implementation of Organ-on-Chip (OOC) instrumentations [8,12,13]. Organ-on-Chip systems describe a microfluidic cell culture device containing perfused chamber(s) for the culture of living cells under physiologically relevant conditions [14].

However, most OOC platforms still rely heavily on endpoint assays lacking the ability to assess different time-points during the test schema. Additionally, chemical labels, such as fluorometric labels, can potentially affect the cellular metabolism and thereby alter the experimental results [15]. Hence, the integration of a real-time read-out system, using label-free techniques, is an essential part in the move toward spatially and temporally resolved measurements with meaningful information.

### 2.3 Transepithelial Electrical Resistance in Skin/Reconstructed Human Epidermis Toxicology

Since potentially harmful compounds also affect skin thickness, the measurement of the thickness was an early parameter to study adverse impacts based on destructive measurements, such as biopsies [16], or non-destructive measurements, such as confocal Raman spectrometers [17] and ultrasound imaging [18]. One of the most conventional approaches and useful techniques for cell culture applications is the measurement of the skin permeability barrier to evaluate the epithelial or endothelial tissue viability and function [19]. Here, transepithelial electrical resistance (TEER) provides a label-free and quick technique to investigate skin integrity. Formally, TEER values describe the electrical resistance across one or several cell layers [20]. 

### 2.4 Disadvantages of Current Transepithelial Electrical Resistance Methods and the Need for Automated Transepithelial Electrical Resistance.

Next to microfabrication approaches [19,21], there are several commercially available external test systems, like the ‘volt-ohmmeters’ (World Precision Instruments, Sarasota, FL, USA), which can be used off the shelf. Due to missing integration into cell culture systems, researchers are transferring the test specimen manually from the bioreactor to the measurement system [22]. This procedure lacks reproducibility and standardization without the option of offering high-throughput capability. Although the next generation of electrode chambers (such as the Endohm chamber) allowed the measurement without removing samples, media as well as reagents had to be applied manually in case of long-term culture and drug studies [23]. Thus, extended studies are often not possible, because old media cannot be removed from the chamber, and there is no option for automated perfusion of fresh media. Summing up, current TEER measurement systems still rely heavily on manual, hand-held systems, affecting the measurement stability and, therefore, the overall reproducibility of the experiment. However, to be considered by the OECD as an accepted test scheme, the assay reproducibility is an essential criterion in the validation process. For this reason, the field of TEER measurements can benefit greatly by integrating an automated TEER monitoring and acquisition system capable of performing programmed test schema with frequent media change.

Herein, we introduce a novel test system for automated monitoring and an acquisition system for TEER measurements, based on the intelligent mobile lab for in vitro diagnostics (IMOLA-IVD) (cellasys GmbH, Kronburg, Germany). An encapsulation design was developed to maintain the commercial EpiDerm™ RhE model cultured on polycarbonate membranes. TEER measurements were applied for studying RhE cultures and integrated into an automated fluidic platform, allowing precise media perfusion and drug application. Additionally, we were able to monitor the extracellular acidification rate (EAR) of the model. The demonstrated study aims at achieving high-content information via providing real-time kinetics, while measurement stability is ensured by integrating an automated fluidic delivery system with an integrated test schema. The presented study demonstrates the design, validation, and application of a noninvasive measurement method to assess skin integrity.

## 3. Materials and Methods 

### 3.1 Intelligent Mobile Lab for in Vitro Diagnostics General Description

The IMOLA-IVD, a lab-on-a-chip assay system, was utilized to automatically monitor the TEER and EAR of L929 murine fibroblast cells and EpiDerm™ reconstructed human epidermal models. The basic operation of the IMOLA-IVD has been described in previous works [24,25]. Briefly, each IMOLA-IVD consists of a power supply, analog and digital modules, and a BioChip with integrated sensors that passively monitor the microenvironment of the cellular model. Standard IMOLA-IVD experiments are automated with a personal computer loaded with the Data Acquisition and Link Application (DALiA) Client 2.0 [26] to control a peristaltic pump and a fluidic network connected to up to six IMOLA-IVD systems. The pump cycles between ON and OFF states. During pump OFF cycles, cells are able to metabolize the nutrients in the medium; during pump ON cycles fresh, nutrient-rich medium is pumped to the cells. 

### 3.2 Design of Modified BioChip-D Encapsulation

**Figure 1:**
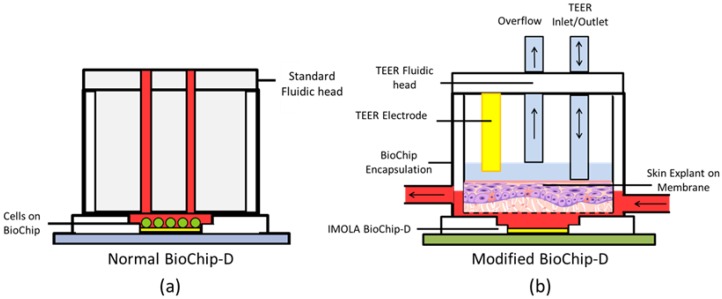
Schematic diagrams: (**a**) Standard BioChip with fluidic head, and (**b**) modified BioChip with transepithelial electrical resistance (TEER) fluidic head. The fluidic system with the medium is highlighted in red. IMOLA: Intelligent mobile lab.

The basic BioChip contains a sensor chip bonded to a printed circuit board and attached to a cylindrical encapsulation, which protects the electrical connections of the chip and supports the cells and their medium. A fluidic head fits directly into the encapsulation, forming a liquid tight seal and creating a reaction chamber with a static micro-volume (~6 µL) to maximize the acidification and oxygenation signals measured by the integrated sensors. This occurs as fresh medium is delivered to cells cultured directly on the sensor surface (Figure 1a). However, in the case of RhE, cells are cultured on top of porous membranes that do not fit onto standard BioChips or support standard impedance measurements with IMOLA-IVD interdigitated electrode sensors (IDES). To overcome these limitations, new encapsulations and fluidic heads were designed to maintain the air-liquid interface (ALI) and support measuring the TEER of RhE using Pro/Engineer Wildfire 4.0 (PTC Inc., Needham, MA, USA). Encapsulations were printed with an Ultimaker 2 (Ultimaker BV, Geldermalsen, Netherlands), using polylactic acid (PLA) and attached to the BioChip with Silastic medical adhesive type A (Corning Inc., New York, NY, USA). The redesigned BioChip encapsulation features an enlarged culture chamber that supports RhE and other membrane-bound cell cultures (Figure 1b). 

### 3.3 L929 Cell and EpiDerm Reconstructed Human Epidermis Preparation and Culture

Transepithelial electrical resistance were recorded from murine fibroblasts (L929) and EpiDerm™ RhE. L929 cells were cultured in Dulbecco’s modified eagle medium (DMEM), supplemented with 10% fetal bovine serum (FBS) and 10 µg/ml gentamycin (Thermo Fisher, Waltham, MA, USA). Cells were cultured according to good laboratory practice (GLP) and stored in an incubator at 37 °C and 5% CO_2_. At 95% confluence, cells were passaged and 100,000 were deposited onto 12 mm Transwell^®^ membrane inserts of pore size = 3 µm (Corning Inc.). Transwells were then placed on six well-plates and incubated in 1 mL of DMEM for an additional 24 h before transferring them to modified BioChips. The L929 cells are a standard cell line in our laboratory and were used for proof-of-concept.

Upon receipt, EpiDerm™ RhE models were transferred to six well-plates and incubated in 5.5 mm over 5 mL of MatTek serum free, long-term culture medium (#EPI-100-NMM-250) (MatTek in vitro life science laboratories). The medium was exchanged every 48 h until transferring them to the BioChips for TEER experiments. 24 h prior to the start of the experiment, the medium was reduced to 0.9 mL and placed on 12 well-plates. Modified BioChip-Ds were sterilized at room temperature in 70% ethanol for 20 min, and rinsed with sterile, deionized (DI) water. BioChips were perfused with unbuffered DMEM for 24 h in order to stabilize temperature before membrane cultures were placed onto the chips.

### 3.4 Description of Automated Fluidic System

**Figure 2:**
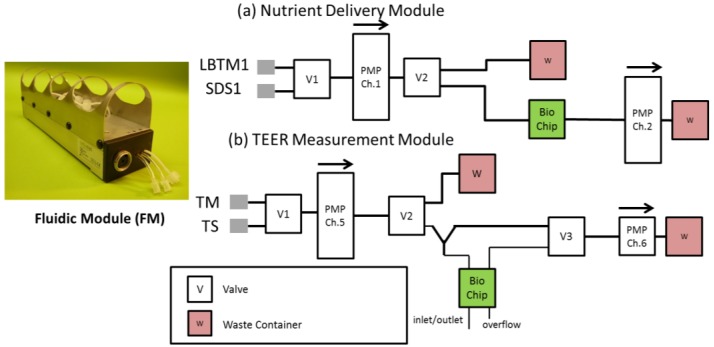
The dual fluidic network consists of (**a**) a nutrient delivery module, capable of transporting cell culture medium to and from the BioChip, and (**b**), a TEER measurement module, which periodically perfuses the apical side of the membrane with phosphate buffered saline (PBS) to measure TEER. PMP: Pump; LBTM1: Low buffer treatment medium; SDS1: SDS Medium; TM: TEER medium, TS: Test substance

An automated fluidic system was devised in order to automatically monitor the TEER of RhE cultures on BioChips over a 48 h period. The system consists of two fluidic modules (FM) connected with Tygon^®^ tubing E-3603 (ProLiquid GmbH, Ueberlingen, Germany) into separate networks. These separate networks supply basal cells with nutrient-rich cell culture medium via the typical ON/OFF IMOLA-IVD pump protocol, and periodically pump phosphate buffered saline (PBS) to the apical side of Transwell^®^ membrane cultures. Block diagrams depicting both of these fluidic networks are shown in Figure 2. The medium delivery module switches between unbuffered DMEM and medium with 0.2% sodium dodecyl sulphate (SDS), which is delivered to the basal side of the membrane to feed the cells. The TEER measurement module is connected to PBS for making TEER measurements or an aqueous test substance that can be used to inoculate the surface of the RhE model.

L929 cells cultured on porous membrane inserts or RhE models were placed on modified BioChips and cultured on-chip in unbuffered DMEM with a predetermined pump cycle for 36 h. For L929 cells, pump ON intervals were five min, whereas pump OFF intervals were varied at five, 10, 30, and 55 min in order to monitor cellular acidification. For RhE models, pump cycles were five min ON, 10 min OFF, five min ON, 25 min OFF, five min ON, 10 min OFF. During the 25-min pump OFF phase, TEER measurements were recorded by pumping PBS into the apical chamber of the membrane insert to bridge an electrical connection between impedance sensor on the chip and the wire electrode in the fluidic head. 750 µL were pumped onto the membrane on the chip at 60 µL/min and were subsequently removed at a rate of 120 µL/min. The TEER measurements were made midway through the 25-min pump OFF period. After a predetermined stable measurement period (47 and 36 h for L929 cells and RhE models, respectively), unbuffered DMEM, supplemented with 0.2% SDS, was pumped into the chips to serve as a positive control. Perfusion with SDS medium continued for at least 12 h to confirm the effects of the control on the measured TEER.

## 4. Results and Discussion

### 4.1 Function of Redesigned Transepithelial Electrical Resistance Encapsulation

Membranes are inserted into the culture chamber, defining the chamber volume to ~170 µL. Medium is perfused via an inlet opening into the chamber formed below the membrane. Pores on the bottom of the membrane allow passive diffusion of fresh nutrients to the basal layer of cells and waste products from the cells. Nutrient-depleted medium is then pumped out of the chamber via an outlet during pump ON cycles (Figure 2). The apical surface of RhE cultures undergoes extended culture periods exposed to ambient air to stimulate differentiation into a stratified layer at the ALI. To measure the TEER of these cultures, redesigned fluidic heads incorporate a bi-directional inlet/outlet valve that periodically dispenses a controlled volume of PBS solution. This solution completes the electrical circuit between a wire electrode that extends into the apical chamber of the membrane insert (see Figure 1b), and a planar IDES electrode on the sensor chip. An overflow valve keeps the TEER electrode submerged in a stable volume of medium and prevents overflow. The TEER of the membrane is recorded continuously for 5 min before the PBS is then removed from the chamber between measurements to maintain the ALI.

### 4.2 Measurement of Extracellular Acidification Rate of Murine Fibroblasts

**Figure 3:**
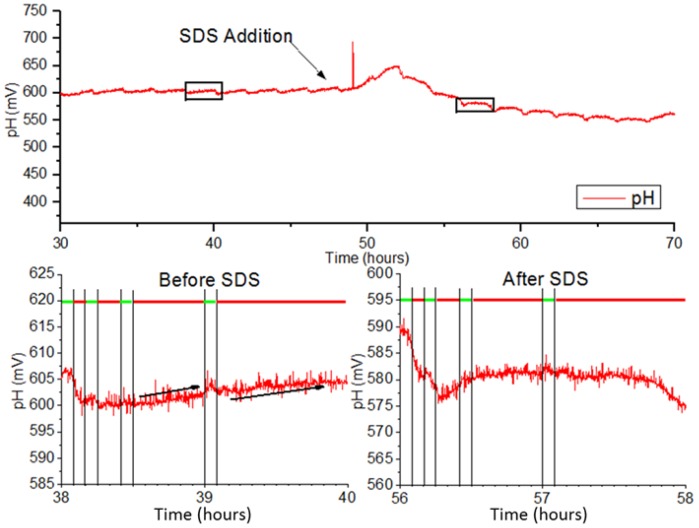
Recorded extracellular acidification (pH in mV vs. time) of L929 cells before and after addition of sodium dodecyl sulphate (SDS). mV: millivolts.

The EAR was monitored using automated measurement protocols with L929 cells immobilized as monolayers on polycarbonate membranes. L929 cells were perfused with unbuffered DMEM at a rate of 50 µL/min. Figure 3 displays the measured pH (in mV) during the alternating pump phases. Red bars indicate pump OFF phases where medium flow is halted, and cells actively reduce nutrients into acidic waste products which are detected by the metal oxide sensors on the BioChip. Green bars indicate pump ON phases. In these phases, the basal side of the membrane is perfused with fresh medium, feeding the layer of cells cultured on top of the membrane. From the chart, pump OFF phases of 55 min produce voltage increases of ~5 mV as the medium becomes acidified, while shorter durations do not generate significant changes in voltage.

### 4.3 Murine Fibroblast Metabolic Reaction to Sodium Dodecyl Sulphate Medium

**Figure 4:**
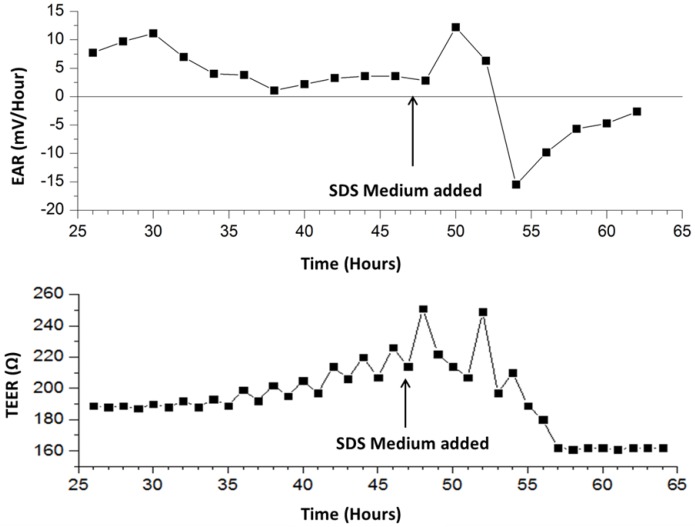
Calculated extracellular acidification rate (EAR) and TEER of L929 cells during 55-min stop intervals; Ω: Ohms.

Once the pump ON cycle concludes, the measured voltage rapidly declines until reaching the baseline (steady state) value. A short delay is present before the decline possibly due to mixing between fresh medium with acidified medium that is already present on the chip. This occurs because the outlet is positioned higher than the inlet. At 58 h, the measured signal drops. This may be due to the rupturing of cellular membranes and release of intracellular contents into the medium. At 56 h, the cells have lysed and therefore produce no waste to acidify the surrounding medium, resulting in a flattening of signal in the 55-min pump OFF phases. Figure 4 depicts the tracked EAR measured during 55-min OFF phases for the duration of the experiment. It can be seen that after SDS application, the EAR spikes and then rapidly drops below zero to indicate the lysis of cells. It then gradually approaches zero until the experiment ends.

*4.4 Transepithelial Electrical Resistance Monitoring of Murine Fibroblast Monolayer*

In addition to monitoring the metabolic rate of L929 cells, TEER values were monitored midway through the 25-min pump OFF period. During the TEER measurement process, values vary as the PBS is pumped into the measurement chamber to bridge an electrical connection for measurement. TEER values begin at immeasurable values due to an open circuit existing between the electrode pair. The wire electrode embedded in the fluidic head becomes immersed in PBS and completes the circuit with the electrode on-chip below the membrane. PBS is allowed to remain on the cells for five min and the pump is switched off for stable monitoring. The PBS is then pumped out of the measurement chamber until the next measurement sequence.

The measured EAR and TEER is plotted vs. time from 25 h in Figure 4. EAR is calculated by linear regression during the pump OFF phases [27]. Results are summarized in Table 1, showing pre- and post-exposure to SDS. 24 h after the start of the experiment, the measured real impedance has stabilized near 190 ohms, while the imaginary impedance is -40 ohms. Because L929 murine fibroblasts are known to maintain a single monolayer in culture, it is expected that the impedance would stabilize at a low level. 8 h post exposure to 0.2% SDS medium (time = 56 h) the real impedance drops by 14.8% in response to cell death mediated by the addition of SDS. The imaginary impedance declines by 6.11% in magnitude. These results demonstrate that the developed encapsulation design and pump modality is capable of making automated impedance measurements across a layer of cells cultured on a polycarbonate membrane insert, enabling transepithelial impedance measurements of stratified cell cultures.

### 4.5 Transepithelial Electrical Resistance Monitoring of MatTek Epiderm Reconstructed Human Epidermis Model

**Figure 5:**
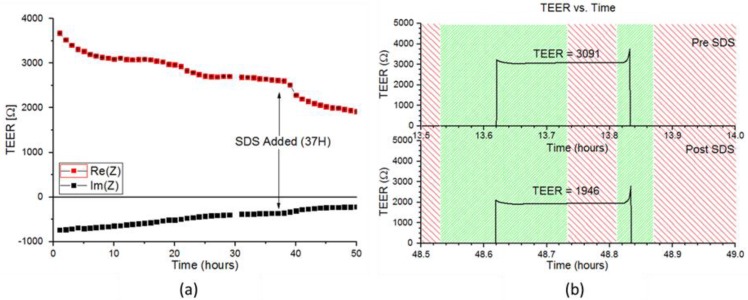
(**a**) TEER values (real part) of the MatTek over time before and after exposure to SDS medium (at time = 37). (**b**) Green areas indicate time periods where fluidic pumping is occurring to either fill or remove PBS from the RhE model. Red spaces indicate places where fluidic pumping in the fluidic head is not occurring. Z: Impedance.

EpiDerm™ RhE were monitored using the same setup as described above. TEER was monitored continuously for ~50 h. For the first 12 h, TEER values remain relatively stable, as seen in Figure 5. After 36 h, cells are treated with 0.2% SDS, and two h after the switch, the average TEER value abruptly drops. Prior to inoculation with SDS medium, the TEER values initially spike, after filling the apical chamber with PBS, then quickly stabilize and remain the same value until the end of the five-min measurement period. At the removal of the medium from the apical chamber, the TEER becomes immeasurable again due to the removal of the electrical bridge.

Changes in the TEER curve are detectable just 2 h after switching to SDS medium. Initially, the TEER value spikes in the same fashion as before inoculation with SDS medium; however, the TEER value steadily decreases until it stabilizes at a much lower value during the measurement period. This causes a reduction in the average TEER measured, which is consistent with cell lysis caused by the addition of SDS. Furthermore, as the time of exposure to SDS medium increases, the magnitude of the initial spike in impedance decreases. The persistence of the initial spike seems to indicate cellular survival, despite exposure to SDS. It is important to note previous IMOLA-IVD studies were performed on simple monolayers of cells and required a relatively low concentration of 0.2% SDS in order to completely kill off cells in a shorter period of time. The delayed lysis of cells can likely be attributed to the increased robustness of cells cultured in the more complex 3D skin model.

## 5. Conclusions

Here, we presented an innovative approach to noninvasively monitor cells cultured in 2D and 3D on porous membranes in a variety of morphologies. Our state-of-the-art BioChip encapsulation design was demonstrated to both sustain and support monolayers of murine fibroblasts, as well the more complex EpiDerm™ human dermal tissue model. Furthermore, a two-part automated fluidic system was devised to handle the delivery and removal of PBS solution into the apical compartment for TEER measurements. Using L929 cells, a standard operating protocol was devised to monitor metabolic rates and barrier integrity by performing periodic TEER measurements. It was shown that the developed procedure maintained cellular viability until inoculating cells with SDS medium as a positive control.

After preliminary tests with L929 cells, the TEER of EpiDerm™ human skin models was monitored. TEER values revealed that skin models could be contained in culture for at least 24 h, and exposure to SDS medium produced a delayed onset of cellular lysis. Higher concentrations can be used in future experiments to improve the response time of the control. Overall, this work stands as a proof-of-concept study that demonstrates the capability of the IMOLA-IVD to be used on complex 3D tissue models as a tool for creating noninvasive automated cellular assays. Notably, this work also presents a proof-of-concept of an automated TEER measurement system that preserves an ALI.

## Figures and Tables

**Table 1:** Impedance values of murine fibroblasts culture on TEER chips.

Time in Culture	Impedance *n* = 9	
	Real (Ω)	Imaginary (Ω)
Pre-SDS exposure	189.66 ± 1.80	−41.89 + 0.93
After SDS exposure	161.56 ± 0.53	−39.33 + 1.00
